# Peripheral blood derived mononuclear cells enhance osteoarthritic human chondrocyte migration

**DOI:** 10.1186/s13075-015-0709-z

**Published:** 2015-08-07

**Authors:** Niina Hopper, Frances Henson, Roger Brooks, Erden Ali, Neil Rushton, John Wardale

**Affiliations:** Division of Trauma and Orthopaedic Surgery, University of Cambridge, Addenbrooke’s Hospital, Hills Road, BC2 0QQ Cambridge, UK; Department of Veterinary Medicine, University of Cambridge, Madingley Road, CB3 0ES Cambridge, UK

## Abstract

**Introduction:**

A major problem in cartilage repair is the lack of chondrogenic cells migrating from healthy tissue into defects. Cartilage is essentially avascular and therefore its healing is not considered to involve mononuclear cells. Peripheral blood derived mononuclear cells (PBMC) offer a readily available autologous cell source for clinical use and therefore this study was designed to evaluate the effects of PBMCs on chondrocytes and cartilage.

**Methods:**

Human primary chondrocytes and cartilage tissue explants were taken from patients undergoing total knee replacement (*n* = 17). Peripheral blood samples were obtained from healthy volunteers (*n* = 12) and mononuclear cells were isolated by density-gradient centrifugation. Cell migration and chemokinetic potential were measured using a scratch assay, xCELLigence and CyQuant assay. PCR array and quantitative PCR was used to evaluate mRNA expression of 87 cell motility and/or chondrogenic genes.

**Results:**

The chondrocyte migration rate was 2.6 times higher at 3 hour time point (*p* < 0.0001) and total number of migrating chondrocytes was 9.7 times higher (*p* < 0.0001) after three day indirect PBMC stimulus and 8.2 times higher (*p* < 0.0001) after three day direct co-culture with PBMCs. A cartilage explant model confirmed that PBMCs also exert a chemokinetic role on ex vivo tissue. PBMC stimulation was found to significantly upregulate the mRNA levels of 2 chondrogenic genes; collagen type II (*COL2A1* 600–fold, *p* < 0.0001) and SRY box 9 (*SOX9* 30–fold, *p* < 0.0001) and the mRNA levels of 7 genes central in cell motility and migration were differentially regulated by 24h PBMC stimulation.

**Conclusion:**

The results support the concept that PBMC treatment enhances chondrocyte migration without suppressing the chondrogenic phenotype possibly via mechanistic pathways involving *MMP9* and *IGF1*. In the future, peripheral blood mononuclear cells could be used as an autologous point-ofcare treatment to attract native chondrocytes from the diseased tissue to aid in cartilage repair.

## Introduction

The overarching goal of repairing articular cartilage lesions is to achieve a functional and viable joint surface in the long term and prevent progression to osteoarthritis (OA) [1, 2]. Defects in adult articular cartilage that do not penetrate the underlying vascularized tissues generally do not heal [3]. Unlike the majority of tissues, cartilage healing does not involve any mononuclear cells as it is essentially avascular. Another issue in cartilage repair is the lack of chondrogenic cells migrating from healthy tissue into local defects. Dogma suggests that chondrocytes in adult cartilage do not migrate in their native environment due to the surrounding highly tensile collagen network resulting in a highly pressurized matrix [4]. However, chondrocyte motility has been reported by a small number of publications [5, 6] which observed that after bovine articular cartilage was injured by blunt impact the defect area was repopulated within 7–14 days by cells that appeared to migrate from the surrounding matrix.

During our previous studies using explants derived from human osteoarthritic articular cartilage cultured in serum-free conditions we have observed the formation of cell monolayers around the explants after 7–10 days in culture [7]. This evidence suggests that human chondrocytes are capable of substantial migration and this action is likely to be initiated either by pre-existing OA damage or by cutting the tissue. The functional role of the migratory cells is not clearly understood [8]. In the study by Seol et al., [6] migratory cells harvested from the cartilage injury area produced more side populations identified by flow cytometry, and expressed lower levels of cartilage extracellular matrix (ECM) genes, such as collagen type II and aggrecan, compared with normal chondrocytes. The migratory cells are thus proliferative and exhibit a phenotype different from chondrocytes [8].

The majority of chondrocyte migration studies in the literature have been conducted by following isolated cell movements on planar surfaces in vitro, but there is also some evidence of ex vivo cell migration from cartilage. Human cartilage explant studies raise the intriguing possibility that matrix injuries resulting in disruption of the collagen network of adult cartilage by extensive cutting [9], collagenase digestion [10] or defect drilling [11] could provide a permissive environment for chondrocyte motility.

Growth factors have previously been reported to enhance current cartilage repair techniques via multiple mechanisms including 1) recruitment of chondrogenic cells (chemotaxis), 2) stimulation of chondrogenic cell proliferation (mitogenesis) and 3) enhancement of cartilage matrix synthesis. Growth factors and cytokines circulate in the peripheral blood and reach articular cartilage through the synovial fluid and several have been proposed to influence chemotaxis in cartilage repair including fibroblast growth factor (FGF), platelet-derived growth factor (PDGF), vascular endothelial growth factor (VEGF), insulin-like growth factor (IGF)1, IL8, bone morphogenic protein (BMP)4, BMP7, transforming growth factor (TGF)β and stromal-derived factor (SDF)1 [12–17]. Traumatized cartilage releases chemoattractive factors for chondrogenic progenitor cells such; PDGF and IGF1, which are known to induce a significant site-directed migratory response [15]. However, at the same time IL1β and TNFα are released and these chemokines inhibit migratory activity which might contribute to the low regenerative potential of cartilage in vivo.

Whilst articular cartilage defects have a limited capacity to repair, cartilage injuries that penetrate the subchondral bone can repair. Shapiro et al. [18] showed that this repair is mediated by the proliferation of cells that invade the defect from the underlying bone marrow and vasculature. This physiological repair response still forms the rationale behind a number of orthopaedic procedures described as bone marrow stimulation techniques [19, 20]. However, whilst this repair capacity is usually attributed to the recruitment of mesenchymal stem cells from the bone marrow or vascular pool, little is known of the contribution of other blood cells to the healing process. One cell type that may be involved is the peripheral blood mononuclear cell (PBMC). These cells can secrete a large number of cytokines [21, 22] and thus, could produce chemoattractants to assist in cartilage repair. Secretome of PBMCs has been previously analysed and reported to enhance wound healing in a mouse model [23]. In addition, articular cartilage regeneration has been reported in the clinics with PBMC therapy using intra-articular injections of autologous PBMCs in combination with hyaluronic acid (HA) in a clinical case study of 5 patients [24] and in a randomized controlled trial of 180 patients [25], with good clinical outcomes.

The aim of this study was to observe whether PBMCs enhance chondrocyte migration without adversely effecting chondrogenicity in order to establish if PBMCs have potential in therapeutic cartilage repair.

## Methods

### Tissues

Human tissue was obtained with full ethical consent in writing to participate in the study from all patients undergoing total knee replacement for osteoarthritis (Cambridge Local Research Ethics Committee No. 06/Q0108/213). The articular cartilage showed characteristic features of OA histopathology, with disruption and fibrillation of the articular surface; however, for the experiments only macroscopically normal-looking cartilage tissue was used. Articular cartilage was harvested from a total of 17 consecutive patients (average age 71.8 ± 7.7 years) from the femoral condyles and tibial plateau using a sterile scalpel blade and diced as finely as possible.

Mesenchymal stromal cells (MSC) were isolated from the infrapatellar fat pad of seven consecutive human donors (average age 71.4 ± 8.3 years). The infrapatellar fat pad was minced using a sterile scalpel blade and placed in medium containing 10 % heat inactivated FBS, penicillin/streptomycin (100 IU/ml and 100 μg/ml), gentamycin (10 ng/ml) and amphotericin B (2.5 μg/ml).

### Primary cell isolation

1. Collagenase digestion: chondrocytes and MSCs were released from the tissue using 0.2 μm filter-sterilized Collagenase A (11088793001, Roche, UK) 0.2 % w/v in Dulbecco’s modified Eagle’s medium containing 10 % heat inactivated FBS, penicillin/streptomycin (100 IU/ml and 100 mg/ml), gentamicin (10 ng/ml) and amphotericin B (Fungizone™ - 2.5 μg/ml) (complete medium). For standard monolayer cultures, the cells were plated on tissue culture plastic at a density of 20,000 cells/cm^2^ and used at passage 3 or less.

2. Explant outgrowth: full-depth slices of articular cartilage were incubated in medium as above in 6-well plates. Cells were allowed to migrate out of the tissue to form monolayers at which point the explants were removed and the cells trypsinised before use in experiments at the same passage number as those derived by collagenase digestion.

### Blood samples

Peripheral blood samples were taken from 12 young (32.9 ± 9.3 years) healthy volunteers using a sterile Monovette EDTA 9-ml tube. The blood was diluted 1:1 with Hank’s balanced salt solution, layered on Lymphoprep™ solution (Axis-Shield, Dundee, Scotland) and centrifuged for 20 minutes at 800 g. The mononuclear cell-rich band was removed, and resuspended in medium supplemented with 10 % FBS and the cells pelleted by centrifugation for 10 minutes at 250 g.

### Cell migration

#### Scratch assay

**Fig. 1:**
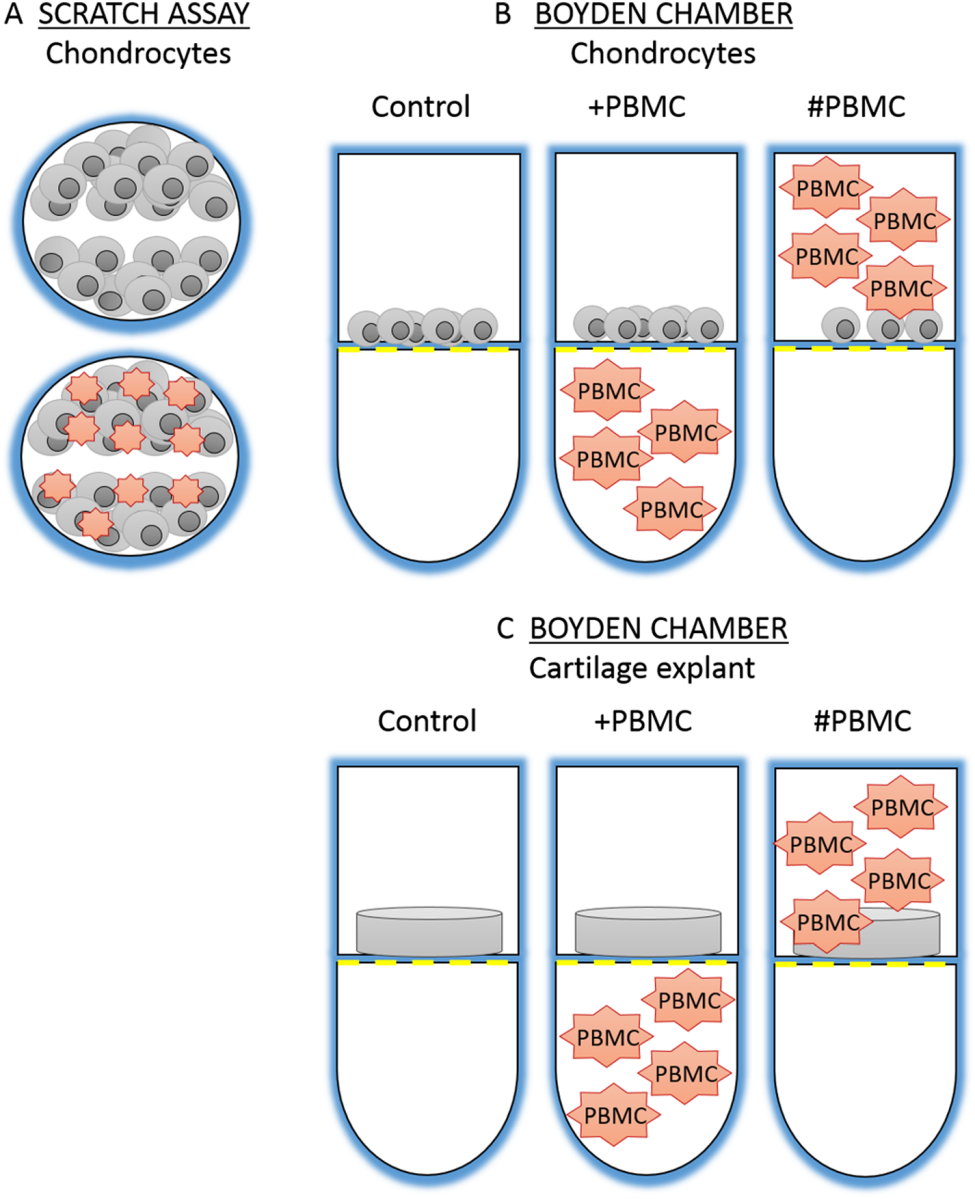
Schematic illustration of migration studies. **a** Scratch assay, **b** Boyden chamber with chondrocytes and **c** Boyden chamber with explants demonstrating the position of cartilage explant and chondrocytes in relation to peripheral blood mononuclear cells (*PBMCs*)

In the scratch assay [26] primary chondrocytes were grown to confluence in a 24-well plate. A thin wound (800 μm) was introduced by scratching the cell monolayer with a sterile pipette tip. Two test groups were compared: 1) chondrocytes alone and 2) chondrocytes together with PBMCs (1:1) to test direct cell-to-cell contact (Fig. 1a). The migration of cells from the wound edge into the wound space was recorded by time-lapse imaging using an Eclipse Ti Nikon microscope and the distance of the gap was measured at three time points and analysed with Nikon Advanced Research Elements 3.21.00 software.

### xCELLigence assay

The migration and chemokinetic potential of the cells was measured using an xCELLigence System RTCA DP real-time cell analyser fitted with CIM plates (05665817001, Roche, UK), which is based on the Boyden chamber model [27]. The CIM plates have 16-well migration units comprising upper and lower chambers separated by a porous (pore size 8 μm) polyethylene terephthalate (PET) membrane in conjunction with microelectrodes. Cell migration (2.0 × 10^4^) was measured with the presence of complete medium but containing 1 % FBS.

In the xCELLigence assay, three test groups were used: 1) chondrocytes in the upper chamber alone (control) and 1 % FBS in the lower chamber, 2) chondrocytes in the upper chamber and +PBMC (as presented on Fig. 1) (1:1) in the lower chamber to test directed cell movement without cell-to-cell contact, 3) chondrocytes in the upper chamber and #PBMC (as presented on Fig. 1) (1:1) together in the upper chamber to test direct cell-to-cell contact effect (Fig. 1b). As a negative control PBMCs in the upper chamber with 1 % FBS in the lower chamber was also recorded. Each experiment was done with four replicates and after equilibration, the analyser was programmed to scan the membrane every 15 minutes. As the half-life of a circulating monocyte has been estimated to be around 3 days in humans the data analysis was performed over 3 days [28, 29].

A similar experimental design was used to analyse if cells can be stimulated to migrate from native human articular cartilage by PBMCs. Full-thickness human articular cartilage explants were prepared 5 days prior to the migration experiment with a 5-mm biopsy punch (Brymill Cryogenic Systems) and cultured in complete cell culture medium. Explants were then transferred to the xCELLigence system under the same conditions as those used for isolated chondrocytes (Fig. 1). The total number of cells migrating was quantified at the end of the study using a cell index (CI) value. CI values are based on impedance measurements providing quantitative information about cell migration through the pores of the membrane. The cell migration rate was measured from the slope of the graph.

### Cell proliferation

In the CyQUANT assay 5 × 10^4^ cells (n = 5) were seeded per well in triplicate in 48-well plates and grown until almost confluent. Following confluence a thin wound (800 μm) was introduced by scratching the cell monolayer with a sterile pipette tip. The cells were stimulated with PBMCs for 24 h, then washed and frozen at −20 °C. The total DNA was quantified using the manufacturer’s instructions (CyQUANT, Thermo Fisher Scientific, Loughborough, UK). Fluorescence (excitation 480 nm, emission 520 nm) was measured on a FLUOstar OPTIMA microplate reader. Similarly, a DNA standard curve was created by diluting lambda DNA in 1 × CyQUANT buffer to give a range covering 1 to 10 ng of DNA in 100 μl of buffer. The standards were also processed and treated similarly to the test samples.

### Cell activity and biosynthesis

Trypan blue exclusion assay was used to determine the PBMC viability in culture at days 1 and 3. In addition, human cytokine array (Proteome Profiler Array, ARY005, R&D Systems, Abingdon, UK) was used to measure the presence of 36 human cytokines secreted by PBMCs in culture at day 3.

### mRNA expression

Digested chondrocytes were cultured with or without non-adherent PBMCs (1:1) for 24 h. After stimulation the PBMC’s were washed away to avoid mRNA from the mononuclear cells in suspension. Chondrocyte mRNA was extracted using TRIzol® reagent (15596–026, Ambion, Paisley, UK) according to the manufacturer’s instructions. The RNA pellet was air-dried and resuspended in 35 μl DNAse/RNAse-free water subsequently, RNA concentration and quality were checked with optical density (OD) 260/280 measurement using a NanoDrop spectrophotometer. Quality was verified by 1.2 % agarose gel electrophoresis using the FlashGel® System (57067, Lonza, Nottingham, UK) and RNA Cassettes (57027, Lonza, Nottingham, UK).

### PCR array

cDNA synthesis was performed with RT^2^ First Strand kit (330401, Qiagen, Manchester, UK) following the manufacturer’s instructions using 540 ng RNA. Human Cell Motility PCR Array (PAHS-128ZA-2, Qiagen, Manchester, UK) was used to identify and compare 84 key genes central to cell movement. A Stratagene Mx3000P® real-time cycler was programmed with HotStart DNA Taq Polymerase activation for 10 minutes at 95 °C and then 40 cycles of 1) denaturation for 15 s at 95 °C and 2) combined annealing/extension for 1 min at 60 °C. The data acquisition was performed during the combined annealing/extension step and the results analysed with RT^2^ Profiler PCR Array Data Analysis software version 3.5 using the Δ cycle threshold (ΔC_t_) method and normalised to the mean of five housekeeping genes used (*B2M*, *HPRT1*, *RPL13A*, *GAPDH* and *ACTB*).

### Quantitative real-time PCR (rtPCR)

Complementary DNA synthesis was performed with SuperScript® VILO™ kit (11754–050, Invitrogen, Paisley, UK) following the manufacturer’s instructions using 2.5 μg RNA. The real-time quantitative PCR reaction was prepared using QuantiFast SYBR Green PCR detection kit (204054, Qiagen, Manchester, UK) together with QuantiTect Primer Assay (Hs_COL2A1_1_SG, Hs_SOX9_1_SG and Hs_IGF1_1_SG) using 50 ng template cDNA on a Stratagene Mx3000P® real-time cycler. The relative copy numbers of target genes were calculated from the standard curve for each gene and normalised to the housekeeping gene, beta-2 microglobulin (Hs_B2M_1_SG). The MMP9 mRNA expression was measured with TaqMan® assay (Hs00234579_m1) according to manufacturer’s instructions using the StepOnePlus™ real-time PCR system.

### Statistical analysis

All samples were collected in four replicates and the data are presented as the mean ± standard deviation (SD) with the significance level set at 0.05. The data were evaluated using Student’s *t* test to determine statistically significant differences with GraphPad Prism 5 software.

## Results

### PBMC stimulation

#### Migration of isolated chondrocytes

**Fig. 2:**
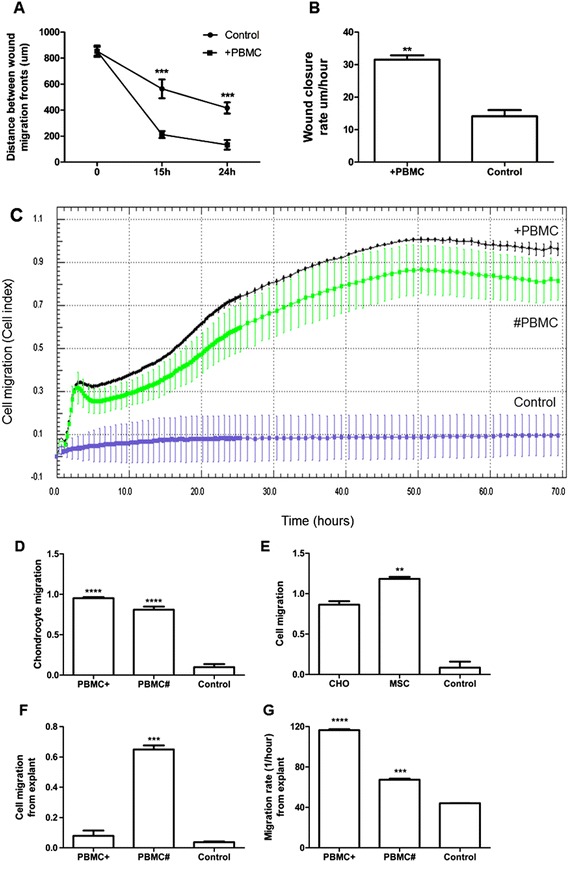
Chondrocyte migration experiment results (n = 4). **a** Scratch assay measuring the distance between the wound migration fronts in a chondrocyte monolayer and **b** wound closure rate in a chondrocyte monolayer scratch assay at the 3-h time point. **c** Representative image of xCELLigence cell migration analysis from four wells (technical replicates) with chondrocytes measured for 3 days evaluating the chemokinetic effect of peripheral blood mononuclear cells (*PBMC*+ in the lower chamber and *PBMC#* in the same chamber). Error bars represent standard deviation and cell index value is based on impedance measurements that provide quantitative information about cell migration through the pores of the membrane measured continuously with microelectrodes. **d** Total chondrocyte migration at day 3 in the xCELLigence assay and **e** xCELLigence assay comparing mesenchymal stroma cell (MSC) and chondrocyte (*CHO*) migration at day 3 (n = 6). **f** Total cartilage cell migration from tissue explant at day 28 in the xCELLigence assay. **g** Cartilage cell migration rate from tissue explant at day 14 in the xCELLigence assay. **p <0.001, ***p <0.0001 and ****p <0.00001

The addition of PBMCs significantly increased chondrocyte motility in both migration assays (Fig. 2). A scratch assay was used to measure chondrocyte migration in a direct cell-to-cell contact model. At 24 h, the scratch width in untreated chondrocyte cultures was reduced by 51 %, whereas with the chondrocytes cultured with PBMCs, it was reduced by 84 % (*p* = 0.002) (Fig. 2a). In addition, the wound closure rate at the 3 h time point was significantly higher (*p* = 0.0009) with chondrocytes cultured with PBMCs (31.5 μm/h) compared to without PBMCs (14.1 μm/h) (Fig. 2b).

To investigate further the direct and indirect effects of PBMCs on chondrocyte migration, a Boyden chamber model was used. In the first 3 h the cell migration rate was highest and the chondrocyte migration peaked at 48 h (Fig. 2c). At the end of the experiment (70 h), the total number of migrating chondrocytes was significantly 9.7-fold higher in the indirect combination with PBMCs (*p* <0.0001) than the negative control (chondrocytes cultured without PBMCs) (Fig. 2d). Chondrocytes in the direct cell-to-cell model increased the migration 8.2-fold (*p* <0.0001).

Total cell migration was compared between chondrocytes and adipose-tissue-derived stromal cells (MSC). MSCs are reported to have very high motility as compared to other adult non-carcinogenic cell types [30]. We have previously shown that the migration of MCSs is also responsive to PBMC stimuli [31] and in this study MSC total cell migration was 37 % higher than that of chondrocytes (*p* = 0.002, Fig. 2e).

#### Migration from cartilage explants

In the cartilage explant study the total amount of cells migrating at day 3 was highest in the co-culture model (# as presented on Fig. 2) a 17.1-fold increase (*p* = 0.001) compared to the control and was increased 2.1-fold in the indirect culture model (+ as presented on Fig. 2) (Fig. 2f). However, the initial cell migration rate from cartilage tissue explant measured during the first 3 h was 2.6-fold higher when explants were cultured indirectly with PBMCs (*p* <0.0001) and 1.5-fold higher (*p* = 0.0002) in a direct co-culture compared to the control (Fig. 2g). In the second negative control (PBMCs in the upper chamber with 1 % FBS in the lower chamber) the xCELLigence system did not detect cells with a radius <8 μm and therefore the cells from peripheral blood did not affect the cell index read-out (data not shown).

### Cell proliferation

**Fig. 3:**
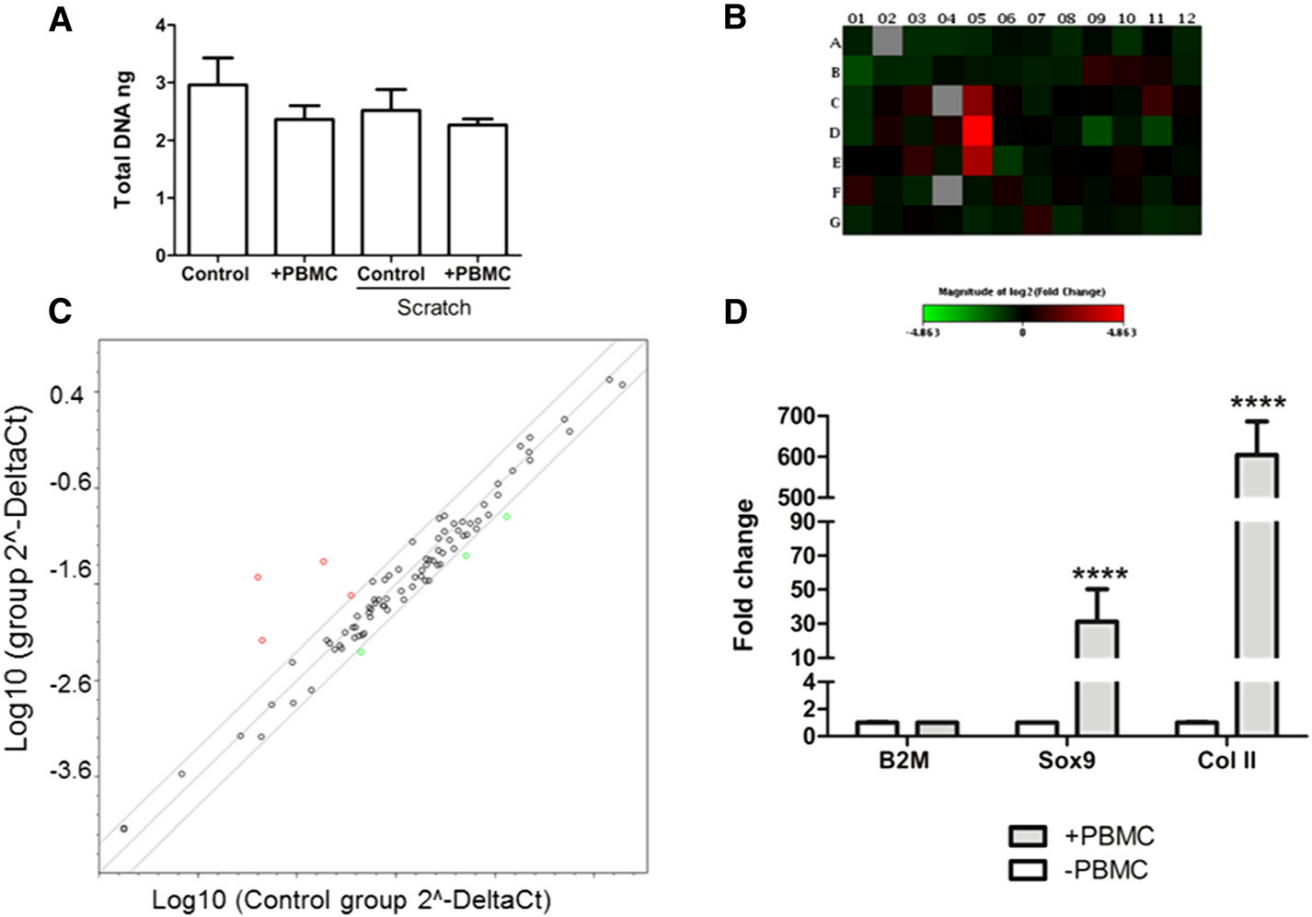
**a** Cell proliferation measured with CyQUANT total DNA assay (n = 5) showing non-significant difference between the test groups. **b** PCR array results showing a change in mRNA levels after 24 h peripheral blood mononuclear cell (*PBMC*) stimulation with a cutoff value of 4. Heat map visualization of 2log2 fold change of the 84 genes in the human cell motility array (*red* upregulated and *green* downregulated). *Grey* shows the genes that were undetermined (no Ct value with a cutoff value of 35). **c** Scatterplot shows up- and down regulated genes and core genes with no change (biological n = 5). **d** Messenger RNA fold change of Collagen type II and Sox9 after 24 h PBMC stimulation (n = 5) *p* <0.00001. All data normalized to B2M housekeeping gene and unstimulated control. In every mRNA expression study chondrocytes in passage 3 were used

In order to evaluate the contribution of cell proliferation to the observed cellular migration, the total DNA was measured in the scratch assay in order to indirectly quantify cell proliferation (Fig. 3a). The scratch lowered the total DNA by 15 % compared to the confluent control well (total 3.0 ng). There was no significant difference between the PBMC-stimulated and non-stimulated test groups (n = 5) in the total DNA amount in either in the control or scratch test group, with −22 % and −23 %, respectively.

### Cell activity and biosynthesis

The cell viability assay demonstrated that 95.4 ± 2.3 % of PBMCs were live in culture at day 1 and 92.2 ± 3.0 % at day 3. In addition, the PBMCs were found to be biosynthetically active secreting 15 different chemokines (C5a, CXCL1, ICAM-1, IL-1β, IL-1ra, IL-6, IL-8, IL-13, IL-16, CXCL10, CXCL11, CCL2, MIF, CCL5, PAI-1) at day 3 in culture (data not shown).

### Quantitative PCR (qPCR)

In order to verify that the migrating cells were maintaining their chondrocyte phenotype and not acquiring a fibroblastic phenotype, qPCR was used to measure two key chondrogenic genes (*SOX9* and *COL2A1*). The mRNA levels for these genes was upregulated by the 24 h PBMC stimulus; *SOX9* (30-fold, *p* <0.0001) and *COL2A1* (600-fold, *p* <0.0001) Fig. 3d.

### PCR array

The mRNA expression of 84 genes involved in the movement of cells including growth factors and receptors important for chemotaxis, genes involved in Rho family signaling and adhesion, and genes encoding components of various cellular projections was quantified with Human Cell Motility RT^2^ Profiler™ PCR Array (Fig. 3b).

Four genes were upregulated by the 24 h PBMC stimulation (n = 5); *matrix metalloproteinase (MMP)9* (29.1-fold change), *integrin (ITGβ)3* (2.2-fold change), *IGF1* (5.8-fold change) and phospholipase D1 (*PLD1*) (9.2-fold change). Three genes were significantly downregulated (n = 5); *MYL9* (−2.6-fold change), *PAK1* (−2.2-fold change) and *CAPN2* (−2.6-fold change) by the 24 h PBMC stimulation as compared to the unstimulated test group (Fig. 3c). The upregulation of *MMP9* and *IGF1* was additionally validated with qPCR (data not shown).

## Discussion

The migration potential of cells is considered important for their integration into host tissue during healing and repair processes as well as for their therapeutic applications. In this study, we have demonstrated that the addition of PBMCs has a profound effect on chondrocyte migration, possibly via *MMP9* and/or *IGF1* mechanisms. In order for a cartilage defect to heal, one key aspect is that the cells that will infill the damaged tissue must migrate to and proliferate at the site of damage. Despite some cell migration potential from cartilage tissue in young experimental animals [32, 33], there is a lack of evidence for chondrogenic cells infiltrating from cartilage tissue into existing defects and even to occupy empty chondrocyte lacunae [34, 35].

It is believed that chondrocytes in adult cartilage do not migrate due to the surrounding highly tensile collagen network resulting in a highly pressurized matrix [4], essentially trapping the cells within the ECM. However, a small number of publications do describe chondrocyte migration in physically damaged tissue [5, 6] or enzymatically degraded matrix [10, 13, 36, 37]. Previous studies have described progenitor cells found in cartilage explants [10, 38, 39] where cells have been allowed to migrate from cut pieces of tissue out onto plastic surfaces. This method may be less damaging to the cells when compared to enzymatic digestion but the impact of differences that the isolation method has on the resultant cell population phenotype is yet not fully understood [39–42]. Enzymatic digestion may negatively affect cellular properties, due to a major alteration of the natural environment of the cells and the removal of cell surface proteins [42, 43]. Our study confirms these observations and we have attempted to minimise any potential cartilage damage by only using macroscopically normal cartilage and preparing the explants using a biopsy punch. However, although stimulation of cell migration by both explant construction and the presence of OA in the joint cannot be ruled out in our study, any clinical application for PBMCs is likely to involve similarly diseased and/or physically damaged tissue.

In order to evaluate the stimulus in both non-direct and direct cell-to-cell contact models, enhancement of chondrocyte migration by PBMC was studied in both a scratch assay and a Boyden chamber model. Our initial observations were that in the scratch assay (direct cell-to-cell contact), there was a significant increase in chondrocyte migration in response to PBMC. Measurements of total DNA in the scratch assay indicated that this observed effect was not due to cell proliferation but was a genuine migration phenomenon. PCR analysis of two key cartilage-related genes (Collagen type II and Sox9) confirmed that these cells were retaining their chondrogenic phenotype and not differentiating towards a more fibroblastic phenotype despite their migratory ability.

To further evaluate the effects of PBMC on chondrocyte migration a Boyden chamber model was used, in which PBMCs could be used to stimulate chondrocytes directly and indirectly. The results show that the total number of migrating chondrocytes was increased both when PBMCs were in direct (8.2 × migration compared to control) and indirect contact (9.7 × migration compared to control). These values are very similar and, indicate that for isolated chondrocytes the physical presence of the PBMC is not the key signalling component of the response and that a secreted factor(s) are likely to be driving the increase in chondrocyte migration.

In contrast, when chondrocytes remained within the cartilage explant, significant cell migration was only detected when the PBMCs were in direct contact with the tissue, with little cell migration seen when the PBMCs were indirectly stimulating the tissue, indicating that direct cell-cell interactions were required to promote migration. The tissue explant model is a better representation of the clinical situation because enzymatically digested chondrocytes in culture have possibly changed their cell surface protein receptors, lost their native conformation together with the extra cellular matrix and they are known to dedifferentiate in a monolayer culture. The extra cellular matrix itself presents a physical barrier for some of the signalling molecules which might explain to some extent the differences in the results comparing the cell migration between chondrocyte culture and cartilage explant in this study. In native environments, cells often navigate in the context of multiple simultaneously presented cues where the ECM environment adds further complexity that could provide conflicting signals to migrating cells if it is not coordinated with chemical gradients [44, 45].

Experiments in young animals have shown that chondrocyte migration affects tissue-engineered cartilage integration by activating the signal transduction pathways involving Src, PLCγ1, and ERK1/2 [46]. In order to begin to investigate the mechanisms underlying the promotion of migration by PBMC in adult OA cartilage, we performed gene array analysis using a human cell motility PCR array. This revealed that the mRNA levels of four genes (*ITGB3*, *IGF1*, *PLD1* and *MMP9*) were significantly upregulated by the 24-h PBMC stimulation in the PBMC/chondrocyte co-culture as compared to the unstimulated test group. Of these genes, *MMP9* (gelatinase B) is an interesting candidate as a key chondrocyte migration regulator. *MMP9* is capable of degrading some components of the ECM and would, in intact cartilage, allow cell migration to occur through the ECM [47]. However, in our study using isolated chondrocytes there is no established ECM to be degraded so a proteolytic role for *MMP9* is unlikely. In support of our study, it has been reported that *MMP9* has a direct paracrine interaction with chondrocytes and leukocytes and this finding could indicate the mechanism for the upregulation of migration by PBMCs [48].

Chondrocyte responses to monocyte/macrophage signals are well described. They lead to catabolic activities of cartilage cells and, thus, contribute to the creation of chronic inflammatory joint disease [49–51]. These catabolic activities are known to increase the secretion of catabolic pro-enzymes such as MMPs and their activated forms. If MMP9 enhances uncontrolled cell migration and accelerates tissue disruption in OA, blocking cell migration might delay degeneration. On the other hand, if the migrating cells function in tissue rebuilding, cell migration should be promoted [8]. Understanding the function of the migratory cartilage cells in vivo at different stages of disease is critical when designing therapies to regenerate the damaged tissue.

Chondrocyte migration has been previously shown to be controlled additionally by growth factors and cytokines. FGF2, IGF1 and PDGF have been shown to promote migration, whilst BMP2, IL1β and TNFα have been shown to inhibit this process [15, 52]. In our study PBMC stimulus demonstrated significant upregulation of *IGF1* in human chondrocytes. IGF1 is an established chondrogenic factor and we have also demonstrated its efficacy in potential regenerative cartilage models demonstrating increases in cell number and ECM deposition within biomimetic scaffolds [53, 54]. IGF1 is also reported to have anti-apoptotic activity in cartilage [55] in addition to triggering chondrocyte mitosis [56]. The population of migratory cells in human articular cartilage tissue have been reported and termed as chondrogenic progenitor cells [57]. These migratory cells from repair tissue have been identified during the later stages of human OA and these cells exhibit stem cell characteristics such as clonogenicity, multipotency and migratory activity [57]. These progenitor cells are exciting targets for cell-based regenerative therapy for joint diseases [58] and in the future it would be interesting to evaluate the effect of PBMC stimulation on chondrogenic progenitor cells.

## Conclusions

The results of this study demonstrate that the presence of PBMCs induces cell migration from articular cartilage and increases both the total number of migrating chondrocytes and the rate of cell movement. This study identifies *MMP9* and *IGF1* as possible regulators for the PBMC-stimulated chondrocyte migration. This novel and encouraging finding both challenges our basic understanding of chondrocyte biology and presents an opportunity for clinical translation. PBMCs are a readily available cell source capable of providing signaling molecules that stimulate chondrocyte motility. Thus, autologous PBMCs could be utilized in a point-of-care treatment to attract native chondrocytes or chondrogenic progenitor cells from the affected tissue to aid in cartilage repair via single or multiple PBMC intra-articular injections.

## Abbreviations

ACTB: actin beta
B2M: beta-2 microglobulin
BMP: bone morphogenic protein
CAPN: calpain
CI: cell index
ECM: extracellular matrix
ERK: extracellular signal regulated kinases
FBS: fetal bovine serum
FGF: fibroblast growth factor
GAPDH: glyceraldehyde 3-phosphate dehydrogenase
HPRT1: hypoxanthine phosphoribosyltransferase 1
IGF: insulin-like growth factor
IL: interleukin
ITGB3: integrin beta 3
MMP9: matrix metallopeptidase 9
MSC: mesenchymal stromal cell
MYL9: myosin, light chain 9
OA: osteoarthritis
PAK1: P21 protein activated kinase 1
PBMC: peripheral blood mononuclear cell
PDGF: platelet-derived growth factor
PET: polyethylene terephthalate
PLCγ1: phospholipase C gamma 1
PLD1: phospholipase D1
RPL13A: ribosomal protein L13a
SDF1: stromal-derived factor 1
Src: proto-oncogene tyrosine-protein kinase
TGFβ: Transforming growth factor beta
TNFα: tumour necrosis factor alpha
VEGF: vascular endothelial growth factor
